# Draft Genome Sequence of Aeromonas cavernicola sp. nov. DSM 24474^T^, Isolated from a Cavern Brook in the Moravia Region of the Czech Republic

**DOI:** 10.1128/genomeA.00227-18

**Published:** 2018-04-12

**Authors:** S. M. Colston, A. Navarro, A. J. Martinez-Murcia, J. Graf

**Affiliations:** a Department of Molecular and Cell Biology, University of Connecticut, Storrs, Connecticut, USA; b Genetic Analysis Strategies S.L., CEEI, Elche, Alicante, Spain; c Area de Microbiología, EPSO, Universidad Miguel Hernández, Orihuela, Alicante, Spain

## Abstract

Species of the Aeromonas genus can be found in numerous environmental milieus, including various water sources, and some species cause disease in animals. We present here the draft genome sequence for Aeromonas cavernicola DSM 24474^T^, a novel species isolated from a freshwater brook within a cavern in the Czech Republic.

## GENOME ANNOUNCEMENT

The genus Aeromonas comprises bacteria that are pervasive throughout the biosphere, occupying a diverse range of niches (1). Some aeromonad species are associated with interactions in animals and humans, ranging from beneficial to pathogenic, while others thrive in aquatic habitats (1, 2). As novel Aeromonas species continue to be discovered, traditional molecular and phenotypic typing methods are being complemented by whole-genome comparisons that provide a better framework for assigning accurate taxonomic identification (3–6). Aeromonas cavernicola sp. nov. DSM 24474^T^ (=CECT 7862^T^, =CCM7641^T^, MDC 2508) was isolated from a water sample obtained from a brook found within a cavern located in the Moravia region of the Czech Republic (7). Initial polyphasic methods indicated that the isolated strain did not cluster with any characterized Aeromonas species, and it has been proposed as the type strain for a novel species (7, 8).

Genomic DNA was extracted using a thermal shock treatment, RNase A digestion, and purified using a silica column. Species identity was confirmed by partial sequencing of the 16S rRNA gene. The sample was sequenced using Illumina’s Nextera XT library preparation and MiSeq sequencer, resulting in 6,049,178 paired-end 250-bp reads. CLC Genomics Workbench v10.1 (CLC Bio, Qiagen) was used to filter, trim, and assemble the reads *de novo*, generating 308 scaffolded contigs, with an *N*_50_ value of 34,187 and an average coverage of 296×. The total genome size (3,887,629 bp) is smaller than the average genome size of aeromonads (4.13 Mb). The G+C content (54.0%) is lower than the average G+C content of aeromonads (60%). Genome annotation was done using the NCBI Prokaryotic Genome Annotation Pipeline, which predicted 3,507 coding sequences and 87 RNA genes. The closest related species, with an average nucleotide identity of 84.06%, is Aeromonas fluvialis (9). This species also has a smaller genome (3.90 Mb).

The genome of A. cavernicola DSM 24474^T^ encodes general type III and VI secretion pathways; these mechanisms may represent important factors in potential host colonization, virulence, and microbe-microbe interactions (10–12). A. cavernicola DSM 24474^T^ also possesses the genes encoding HipA and HipB, factors that have been shown to be important for persistence (dormancy) and multidrug resistance in Escherichia coli (13). Genome analysis also showed the potential breadth of this strain’s metabolic diversity for utilization of numerous carbohydrates or compounds as sources for carbon, nitrogen, or energy, including amino sugars and organic sulfur. DSM 24474^T^ also contains more than 80 predicted mobile elements, including insertion sequences from the IS*1* and IS*5* families, and at least four loci that contain phage-related elements but no complete prophages (14, 15). Sequence analysis also revealed a locus for clustered regularly interspaced short palindromic repeat (CRISPR)-Cas3 system genes, which are found in various *Proteobacteria* (16). The genome sequence of A. cavernicola DSM 24474^T^ gives us insight into an environmental isolate that may be able to adapt to niches beyond its original isolation source.

### Accession number(s).

This whole-genome shotgun project has been deposited at DDBJ/ENA/GenBank under the accession number PGGC00000000. The version described in this paper is version PGGC01000000.
